# Maintaining primacy of the patient perspective in the development of *patient-centered* patient reported outcomes

**DOI:** 10.1371/journal.pone.0171114

**Published:** 2017-03-03

**Authors:** Rochelle E. Tractenberg, Amanda Garver, Inger H. Ljungberg, Manon M. Schladen, Suzanne L. Groah

**Affiliations:** 1 Collaborative for Research on Outcomes and –Metrics, Georgetown University Medical Center, Washington, D.C., United States of America; 2 Department of Neurology, Georgetown University Medical Center, Washington, D.C., United States of America; 3 Department of Biostatistics, Bioinformatics & Biomathematics, Georgetown University Medical Center, Washington, D.C., United States of America; 4 Department of Rehabilitation Medicine, Georgetown University Medical Center, Washington, D.C., United States of America; 5 MedStar National Rehabilitation Hospital, Washington, D.C., United States of America; 6 MedStar Health Research Health Institute, Hyattsville, Maryland, United States of America; University of Liverpool, UNITED KINGDOM

## Abstract

The objectives of this study were to describe and demonstrate a new model of developing patient reported outcomes (PROs) that are patient-centered, and to test the hypothesis that following this model would result in a qualitatively different PRO than if the typical PRO development model were followed. The typical process of developing PROs begins with an initial list of signs or symptoms originating from clinicians or PRO developers; patient validation of this list ensures that the list (i.e., the new PRO) is interpretable by patients, but not that patient perspectives are central or even represented. The new model begins with elicitation from clinicians and patients independently and separately. These perspectives are formally analyzed qualitatively, and the results are iteratively integrated by researchers, supporting clinical relevance *and* patient centeredness. We describe the application of this new model to the development of a PRO for urinary signs and symptoms in individuals with neuropathic bladder, and test the hypothesis that the two processes generate qualitatively different instruments using a national validation sample of 300 respondents. Of its 29 items, the new instrument included 13 signs/symptoms derived from existing clinical practice guidelines, with 16 others derived from the patient/focus groups. The three most-endorsed items came from the patients, and the three least-endorsed items came from clinical guidelines. Thematic qualitative analysis of the elicitation process, as well as the results from our national sample, support the conclusion that the new model yields an instrument that is clinically interpretable, but more patient-centered, than the typical model would have done in this context.

## Introduction

International health agencies, federal funders, and patient advocacy groups have long advocated the involvement of patients in clinical research. For example, the World Health Organization (WHO) established a Patients for Patients Safety program in 2005, with the vision “to engage, empower, encourage and facilitate patients and families to build and/or participate in (a) global network advocating for, and partnering with health professionals” focusing around safer and more people-centered health care services. [1] Although the WHO program was developed specifically to improve patient safety worldwide, the program also highlighted the importance of the patient perspective in health care. Over 25 years earlier, Read et al.[2]had argued that”…meaningful measures of health-related quality of life must be used to evaluate health care interventions.” [2] Whether originating in safety, advocacy, or health-related quality of life, there is agreement about the importance of including “measures of overall health” in the evaluation of health care interventions, because “*overall health is the object of health care*.”[2] [3]

Departing slightly from these earlier perspectives on patient engagement in clinical research, patient-reported outcomes (PROs) were defined by the US Food and Drug Administration (FDA) to be “any report of the status of a patient’s health condition that comes directly from the patient, without interpretation of the patient’s response by a clinician or anyone else.” [4] This definition (the final version of PRO guidance first drafted for comment in 2006 [4], see p. 1) signals the interest of FDA regulators in a focus on the patient’s experience with, or perspective on, interventions within the agency’s jurisdiction. The suggestion that regulation and labeling should (also) include patient perspectives only solidified the importance of the PRO in the biomedical, and particularly the clinical, research literature base. In 2010, the Patient-Centered Outcomes Research Institution (PCORI) was established by the US Congress, specifically to promote (and fund) research that is “…designed to improve patient care and outcomes through patient-centered comparative clinical effectiveness research.” [5]

Since the milestones of the FDA’s definition of the PRO^4^ and the establishment of PCORI in 2010, [5] there has been a notable increase in peer-reviewed publications relating to how to engage patients and other stakeholders in clinical research[6] and about other methodological considerations relating to comparative-effectiveness research. [7] Several models of how to engage patients throughout the research process have been published recently. [8–13] Among these recent publications is a conceptual framework intended “…to help guide the field (of clinical research) toward promising practices in research engagement”. [14] However, in addition to these manuscripts outlining “how to” obtain patient and stakeholder perspectives on research, there have also been others outlining the challenges that specific disciplines may face in engaging patients in clinical research within these fields (e.g., radiology,[15, 16] emergency medicine,[17] and neurosurgery[18]).

As appreciation for the importance of the patient perspective in clinical and health research has grown in the United States, the difference between a patient-reported outcome and one that is also patient-*centered* is often blurred. For example, Chen et al.[19] define “patient-centered outcomes research” as integrating “data from patients *and other stakeholders* to improve health care delivery and outcomes and guide health care decisions”[19]; here “patient-reported” is used as a synonym for “patient-centered,” although the FDA’s definition of a PRO can be interpreted as more specific to the patient him-/herself. While Chen et al.[19] incorporate the patient and other stakeholders explicitly in their definition of patient-centeredness, they do not provide details on how to achieve authentically *patient-centered* (PC) outcomes. Similarly, Frank et al. [20] state that, “(t)aking a patient-centered approach … is intended to produce research that looks beyond the questions and measures found ‘under the lamp post,’ within the researcher’s field of view.” [20] This perspective reinforces the idea that the “researcher’s field of view” does not necessarily encompass the patient’s experiences, but also does not provide details on how patient engagement or patient-centered outcomes can be developed–achieving research that “looks beyond …the researcher’s field of view” and also substantively contributes to the knowledge and practice of the field.

To address this gap, we argue that “patient-reported” and “patient-centered” are not synonymous, echoing the position originally advanced by Basch et al. [21] and Lasch et al.[22] A patient-*reported* outcome can be a patient’s report of *any* outcome, sign, or symptom that is perceived by the patient- whether it originates with him/her or with the individuals (clinicians, researchers, or clinician-researchers) who developed the outcome. By contrast, a patient-*centered* patient-reported outcome is the patient’s report of an outcome that *represents the patient’s experience specifically*–originating with the patient and not with clinicians, clinical researchers, or other stakeholders. With both PRO and patient-centered PROs (PC-PROs), the patient *is* doing the reporting; the concepts of PRO and PC-PRO differ in terms of the *origins* of what the patient is reporting about. Hence, PC-PRO is a subtype of PRO. Specifically, a patient-centered PRO (PC-PRO) is a type of PRO that explicitly maintains the primacy of the patient’s, vs. the clinician’s or researcher’s, perspective on the patient’s health status, condition, or experience. This distinction is consistent with Lasch et al.[22] and Basch et al.[21].

**Fig 1:**
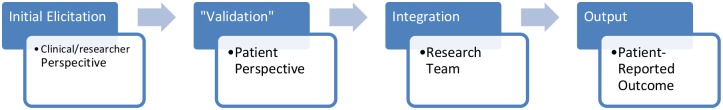
Typical paradigm for developing patient-reported outcomes.

Asking patients to report on clinically-relevant or researcher-identified signs and symptoms is generally consistent with the FDA definition of a PRO, but it tends to maintain the perspective of what is clinically relevant because the clinical perspective first identifies what should be rated, and then patients rate their experience of that (and only that) set of signs and symptoms, typically using the rating scale specified by clinicians. This dominant paradigm, shown in Fig 1, is not consistent with the high-fidelity qualitative approaches to PRO development outlined in Lasch et al.[22], but is *common* for developing a patient-*reported* outcome.

The usual starting point for the development of new patient-reported outcomes is the clinical perspective of the target medical issue or context. Expert clinical opinion is traditionally obtained by interview, focus group, or as a result of an expert panel convened to articulate diagnostic or other criteria, which might be published as position papers or consensus practice guidelines. Although Chen et al.[19] correctly note that the development of a PRO begins with “direct patient input,” *preparation* for focus groups (or other methods to elicit information from patients) is not discussed. While Lasch et al.[22] note that qualitative approaches to the development of a new PRO include the collection of data via focus groups or interviews (among other methods), the interview structure/questions *originate with the investigators* (clinicians or researchers developing the instrument and/or planning the clinical study); patient input therefore generally takes place at the second step in Fig 1 (“validation”). That is, the essential preparation for focus groups or interviews with patients often represents—and maintains—the clinical or investigator perspective. Patient *report* and patient *centeredness* are conflated in this approach; for example, Chen et al.[19] describe patient validation as necessary “…to ensure that the instrument is clearly understood *and* that scores reflect the intended patient voice”.[19]

Fig 1 therefore represents a logical and systematic process of developing patient-reported outcomes that begins with an initial list of signs or symptoms—which is crucial in order to structure the interaction (e.g., interviews and focus groups) with patients or other stakeholders; these typically arise from clinicians or PRO developers (“initial elicitation,” Fig 1). This initial list is then sent/presented to a representative sample of potential patients for their “validation.”[19, 23] In this paradigm, the validation typically derives from/consists of ensuring that the patients *can* report on whatever the clinicians have specified; in some cases, these patients give feedback as to the clarity of either the signs or symptoms or the ratings that are expected.[19] Once patient input is reconciled with (or integrated into) the original clinical perspective, with or without further iteration with clinicians and/or patients, then the patient-reported outcome is ready for pilot testing or further validation.

Recent publications in patient reported outcomes and health-related quality of life stress the importance of partnership between/engagement with researchers *and* stakeholders,[7, 14, 20, 24–26] and Fig 1 is consistent with this emphasis because it shows that nothing results from the development process that did not go through the patients first. The challenge this model presents is that the origins of what patients are reporting on is clinician-, and not patient-, *centered*. As such, PROs developed following the paradigm in Fig 1 may present difficulties in the identification of “response” or “responders,” because the treatment “benefit” constituting a “response” would be defined by the clinical, and not the individual patient’s, perspective.[27]

**Fig 2:**
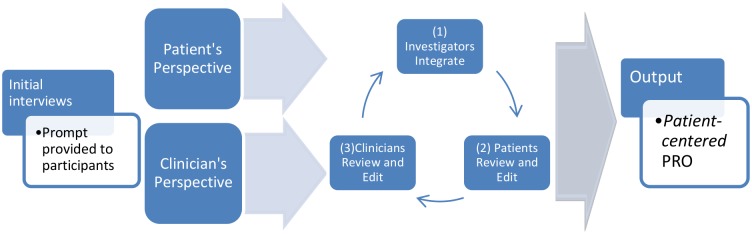
Framework for developing a patient-*centered*, Patient Reported Outcome (PRO).

Like Lasch et al.[22] and Basch et al.,[21] we define a patient-*centered* outcome as one type of PRO that starts with a focus on what the patients experience or what they prioritize about their experience, and not the patients’ report on what clinicians or investigators prioritize about the patients’ experiences. Unlike Lasch et al.[22], however, the approach shown in Fig 2 is iterative, with independent streams of input from patients and clinicians being integrated after inquiries (e.g. interviews and focus groups) have generated each source of input.

The model for PC-PRO definition shown in Fig 2 is achieved by executing the following steps.

1. Assemble an initial list of signs and symptoms based on literature review and independent discussions/experience with patients, clinicians, and researchers. Using this artifact as the point of departure, structure scripts that can support consistent focus groups and interviews around the phenomenon of interest.

2. Gather the independent perspectives on, and experiences of, the phenomenon of interest. Instruct all participants, clinicians and patients alike, that the output (the PRO) will be a list of signs and symptoms that reflect the patient’s perspective, to align clinicians and patients to that perspective. Ongoing formal qualitative analysis of interviews and focus groups separately from each perspective ensures the timely identification of saturation (further discussion or interviews cease to add new details or items to the existing list of signs and symptoms).

2a.1. Employ Consumer Experts (CEs, representatives of the target patient population or experienced caregivers) in the conduct (facilitation, transcription) of patient focus groups. The initial symptom list is a starting point and the CEs are the first patient perspectives elicited on this list to begin its evolution. The CEs will be conducting focus groups and for this, an overall script is required. Once the CEs finish their initial modifications to the symptom list and the focus group structure/scripts, they are able to begin focus groups.

2a. 2. Conduct a series of “pilot” focus groups to understand the functioning of this initial script and to specifically obtain input on how it should evolve to optimize the next series of focus group conversations. In all focus groups, the script is shared to provide cues and context for all participants as they respond to the script and to one another’s experiences. All focus groups are recorded and transcribed. CEs participate in ongoing discussions of focus group results relating to the symptom list and the script/presentation, making any modifications suggested by participant responses (e.g., to clarify prompts and the descriptions of signs or symptoms).

2b. Engage clinical experts in focused discussions and/or interviews to elicit their experiences during care of patients with the phenomenon of interest and their perspectives on the patients’ experiences. Detailed meeting notes, circulated to the participating clinicians between meetings for comment, serve as the content for developing and evolving the clinician-prioritized list of signs and symptoms. Alternatively, clinical experts can be engaged to conduct focus groups in a fully analogous method to that for the patients (2a.1-2a.2).

3. Iteration: reconciling and synthesizing patient and clinician perspectives.

Once both patient and clinician perspectives reach saturation, code the final results with at least two independent coders and a formal qualitative method. Iterative integration of the two perspectives by the research team begins with the identification and removal of duplicate items (i.e., that were identified by both perspectives; and that emerge within one group as two versions of the same sign or symptom). During this process, ambiguities that the investigators perceive in the results from either group are also uncovered, and all plausible interpretations of items are generated—for reconciliation/revision by the clinicians and patients. This is an iterative process whereby any changes made to the “unified results” are then presented to the clinicians for evaluation of whether the changes resulted in different meanings or a loss of specific items or nuance/meaning. The integration process focuses on identifying and eliminating duplications, and identifying and clarifying any vagueness so as to promote the most interpretable responses to any item in the resulting instrument. Additionally, when materials are returned by investigators to either of the other two groups, they are accompanied by an inquiry along the lines of, “is there anything **missing** from this list?”, to ensure/confirm that the saturation that initiated this integration step was correctly identified. The iterative integration stage ensures that the perspectives of both groups are represented in the integrated results. When the unified list stops changing at the integration step, the reconciliation and synthesis are complete and the result is the beta version of the new instrument.

4. Test the beta version of the instrument with a large, representative national sample of target population patients.

Several differences between the paradigms in Figs 1 and 2 are worth noting. First, the initial elicitation, the prompt to gather perspectives on the clinical topic of interest is shown explicitly in Fig 2 –it is *implicit* in Fig 1 (also, see Chen et al.[19]). That is, Fig 2 shows that inquiry in the area of interest must be framed, and prompts developed (scripted), to solicit the perspectives of both patients and clinicians, independent of one another. We also separate out “the investigators” from the clinicians whose clinical experiences and expert input we seek in parallel with the perspectives and lived experience of the patients. Instructions to both types of contributor should be explicit that their focus should not necessarily include what the “other group” might think about what they/their group generated. This independence approach ensures that differences and nuances in viewpoints and opinions between patient experts and clinical experts will be accounted for in the analysis of their respective focus groups and interviews. All participants should be informed of the simultaneous and independent processes by which “their group” will be generating its contributions to the structure of future focus groups in the development process (see Methods).

The remainder of this manuscript describes an example of PC-PRO development that uses the method shown in Fig 2. To do so, we explored the hypothesis that starting with, and prioritizing, the clinician/researcher perspective (as shown in Fig 1) leads to a *clinician/researcher-centered* patient-reported outcome, whereas the alternative approach (Fig 2) leads to a qualitatively different (*patient-centered)* patient-reported outcome. Although previous work has articulated the difference between patient-centered and patient-reported outcomes, this is the first study to our knowledge that tests the hypothesis that the models in Figs 1 and 2 will lead to qualitatively different instruments.

The domain in which this research is conducted is urinary signs and symptoms for people with bladder dysfunction caused by neurologic damage (*neurogenic/neuropathic bladder*, NB). This qualitative study was carried out as part of a larger initiative to develop a PC-PRO instrument in the domain of urinary signs and symptoms in NB. Existing patient-reported instruments for the domain focus on non-NB populations include women with recurrent UTI, women with urinary incontinence or men with prostatic hypertrophy–so existing instruments are not applicable, not even for developing an initial list of signs and symptoms. Clinical practice guidelines are also not useful to develop the initial elicitation scripts, because “urinary tract infection” (UTI) is common for persons with NB, but it is acknowledged in existing guidelines that people with NB have a different UTI experience than do those without NB.[28–30] Therefore, there is a need for a new instrument that is specific to individuals with NB.

## Methods

Approval for all parts of this study was received from the MedStar National Rehabilitation Hospital Institutional Review Board (IRB # 2013–187). The research participants in this case were the focus group participants recruited from the region and nationally, and their informed consent to participate was obtained verbally via a Join Me [31] link (audio). CEs were contracted to be trained and participate as focus group facilitators. Clinicians who participated in face to face focus group meetings, moderated by a clinician with extensive experience in structured interviewing, were informed of the purpose of their participation and that they were free to not participate if they chose. This set of consent procedures was included in the protocol approved by the IRB.

Study investigators developed the model for PC-PRO definition shown in Fig 2, and followed it by executing the following steps.

### 1. Initial assembly of items

Investigators (SLG, MMS) who have worked in this research area over the past 10 years as a clinician (SLG) and as researchers (MMS, SLG) assembled a preliminary list of urinary symptoms based on literature review and discussions with patients, clinicians, and researchers. This list was the point of departure, from which scripts were structured by five consumer experts (CEs) for their use to moderate focus groups among patients around the phenomenon of urinary symptoms in neuropathic bladder.

### 2A. Focus groups and focused meetings

Using a phenomenological approach, recommended as best practice by Lasch et al.,[22] we conducted focus groups with patients (facilitated by trained CEs) and focused meetings of clinicians (facilitated by an experienced clinician)–independently and simultaneously—to obtain a range of individual perspectives and experiences. Patients were not included in clinician focus groups, and clinicians were not included in patient focus groups; all participants were instructed that the objective was to derive an instrument that reflects the patient’s perspective on urinary symptoms. Recruitment of all patient focus group participants was achieved through IRB-approved general invitations to the local neuropathic bladder community (individuals who have expressed interest in participating in the ongoing research of our team), as well as on Facebook, and national advocacy networks. Focus groups were joined by local and national participants who provided verbal consent as approved by our IRB.

We hired five CEs to conduct the focus groups and to support our efforts to collect and integrate patient perspectives on the urinary signs and symptoms of neuropathic bladder. The CE group was composed of two individuals with spinal cord injury, one adult with spina bifida, and two individuals who were a parents/caregiver of minor and adult children with spina bifida. A member of the research team with expertise in qualitative methods and online learning [MMS], who did not interact with the clinical experts involved in the project, served as the trainer and of, and an ongoing resource for, the CEs. CEs were oriented to the objectives of the research project, completed institutional requirements for training in human subjects research, and received further training in online focus group moderation. Two CEs also chose to complete advanced training in qualitative analysis. A member of the research team with expertise in qualitative methods and online learning [MMS], who did not interact with the clinical experts involved in the project, served as the trainer and of, and an ongoing resource for, the CEs. All interactions with CEs, including focus groups, took place as online meetings over Join.Me.[31]

These five CEs conducted a series of three “pilot” focus groups to determine the functioning of the initial script and to obtain input from patients on how this script needed to evolve further in order to optimize the next ten focus groups.[32] All focus groups were led by two CEs, one acting as the moderator with a second CE taking notes. All focus groups were recorded (audio and video) and transcribed.

The focus group script was shared as a Microsoft PowerPoint presentation to provide cues and context for participants as they responding to questions in the script and to one another’s experiences. Moderators member-checked each identified symptom with the participant who was describing it during the course of the focus group. Each focus group took place online with screen sharing. One of the two moderators who managed each group would write a synopsis of the symptom in the shared place for verification and elaboration by the participant offering it and other participants.

The two CEs who had received further training in qualitative methods began to code the focus groups. Using Grounded Theory Methods,[32] these two CEs collaboratively reconciled the codes, categories, and themes they identified in participants’ shared experiences and perspectives and generate a preliminary, global symptom list. This list was member-checked against the impressions of the full group of CEs. CEs met weekly to discuss the symptom list and the script/presentation, making any modifications suggested by participant responses (e.g., to clarify prompts and the descriptions of signs or symptoms). This iterative script evolution is consistent with the principle of emergence in qualitative inquiry.[32] After the first three focus groups, the script did not change further.

Because this was the first time collecting input from clinicians within the model shown in Fig 2, we did not create a parallel focus group series for the clinicians. Instead, one of the investigators (a clinical expert in the domain who also has extensive experience conducting structured interviews, SLG) conducted a series of focused discussions with clinical experts in urology, neuropathic bladder, spinal cord injury, and spina bifida to elicit their experiences and perspectives on urinary symptoms experienced by their patients with neuropathic bladder.

The clinician group participants provided verbal consent as approved by our IRB. This group included three individuals: two Urologists (one MD, one MD-PhD), one Urology Fellow (MD). These participants were in addition to the facilitator, a Physical Medicine & Rehabilitation physician (MD), who is a member of the research team (SLG). These clinicians were invited to participate via email and meetings were held in person; these four clinicians have been collaborating on research in urinary symptoms in persons with neuropathic bladder for many years (although the fellow has only joined the group within the previous year). Five focused meetings were held with the clinician group to capture their perspectives, grounded in their experience caring for patients with NB together with signs and symptoms derived from clinical practice guidelines for urinary tract infection diagnosis.[33] Detailed meeting notes, which were circulated to the participating clinicians between meetings for comment, yielded the consensus, clinician-prioritized list of urinary signs and symptoms in neuropathic bladder.

### 2B. Data analysis

#### 2B.1. Patients’ perspective

Once the CEs had completed three focus groups, two CEs who had received further training in qualitative methods began using the NVivo10[34] software package to code the focus group results. Using Grounded Theory Methods [32], these two CEs worked to collaboratively reconcile the codes, categories, and themes they identified in patient participants’ shared experiences and perspectives.

Member checking occurred throughout the process of the focus group script development, as well as in all focus groups, and the transcription and coding of the results from each perspective into the beta version of the instrument. Preliminary findings were fed back to the larger CE group to verify interpretation of what the focus group participants had shared. Ultimately, the five CEs collaboratively generated a single set of results–based on coding by the two trained CEs—from all 13 of the focus groups. This set of results identified symptoms representing participants’ shared experiences, as well as describing the symptoms in a way consonant with participants’ expression of them.

#### 2B.2. Clinicians’ perspective

Because the results from the clinicians’ meetings were notes, and not transcriptions, coding was not required. The experienced clinician (SLG) member-checked during each focused meeting and another member of the research team (AG) took notes at each meeting. They collaboratively generated a single set of results based on the notes from each meeting and all comments/input from clinicians to the circulated notes. Once the clinician group had nothing to add to their consensus document, it was deemed ready to advance to the next phase in the process of iterative integration with the reports from the patients’ focus groups. Member-checking was incorporated throughout the focused meetings with the clinicians, as well as indirectly through the sharing of all clinician meeting notes among the clinicians.

### 3. Iteration: Reconciling and synthesizing patient and clinician perspectives

Once both patient and clinician perspectives reached saturation, iterative integration of the two perspectives by members of the research team focused on identification and removal of duplicate items. All changes made to the “unified results” were then presented (separately) to the clinicians, and the CEs, for evaluation of whether the changes resulted in different meanings or a loss of specific items or nuance/meaning. The iterative integration stage ended with the CEs to ensure that the perspectives they heard in their own focus groups remained clearly represented in the integrated results. Once the unified list stopped changing at the integration step, the result was the beta version of the new instrument, the USQ-NB.

### 4. Validation of the instrument

Once a beta version was created, we were able to identify which items arose from the patients; items that are included in the clinical practice guidelines were also identifiable from the beta version of the instrument. With this labeling, we would be able to test our hypothesis that the PC-PRO derived following the new model would differ qualitatively from the instrument we would have had if we had started with the symptom list comprising the practice guidelines and not sought independent items from patients. This would be accomplished by testing the beta version of the instrument with a large, representative national sample of patients.

This was achieved by advertising the need for responses on this new instrument by email and through Facebook, and with the assistance of the national advocacy networks in spinal cord injury and spina bifida. All of these outreach and recruitment efforts were advertisements seeking respondents with NB to visit the URL we established for data collection. Within six months, 300 independent and national responses had been obtained from individuals with spinal cord injury and our analyses focus on these responses (the recruitment continued to accrue responses from this patient group, as well as from spina bifida patients and their caregivers, but these results are beyond the scope of this study).

To determine whether our method (Fig 2) resulted in a qualitatively different instrument than would have resulted if we had followed Fig 1, we utilized the clinical practice guidelines (CPGs) set out by the Infectious Diseases Society of America.[33]. Our assumption was that if we had followed Fig 1, these CPGs would have represented our instrument and we would have obtained patient input, through focus groups, on their relevance, impact, severity, and/or frequency. All items on the beta version of our instrument that were not included in the CPGs were then recognizable as “patient-perspective” and not “clinical perspective” in origins. This then enabled us to determine if the approaches in Figs 1 and 2 result in PROs that differ in qualitative (or quantitative) ways. We evaluated the national sample responses to each item to determine whether respondents endorsed items deriving from the clinical guidelines differently from those derived specifically from the patient perspective.

## Results

There are two sets of results presented here: the first outlines our findings representing the qualitative differences in the development process arising from patient*-centered* vs. patient *reported* (clinical/research centered) perspectives on developing this instrument (first from the development of our scripts for the focus groups, then from the focus groups themselves). The second set of results outline the differences in what the final instrument would have captured if we had used Fig 1 (using the clinical guidelines exclusively) vs. Fig 2 (integrating clinical and patient perspectives) to develop the PRO.

### Results: Development of the instrument: Emergence of the focus group script

The table that follows presents an early point in the emergence of the final focus group script from thematic analysis (2.B.1, 2.B.2 above) with the integration by the research team. Table 1 captures the evolution of the script that was used in patient focus groups, derived from the initial symptom list, script elements, and prompts proposed by clinical experts as distilled by the research team (first column), juxtaposed with these same elements as initially (independently) proposed by our patient experts (CEs, second column). These responses have been sorted, but have not been edited. All of the qualitative data are presented in Table 1.

**Table 1:** Comparison of perspectives of clinical and consumer experts with the final patient focus group script elements, organized by general domains of queries.

Clinical experts	Patient Experts	Final elements
**Domain: Symptoms**	**Domain: Symptoms**	**Integrated Domain: Triggers (Symptoms) that lead to patients seeking care**
How do your urinary symptoms usually start. How do you describe, in terms of personal experience, acute UTI? At what point do you contact your care provider be contacted?	How did your symptoms start? Is this how they usually start or was it different? Do you notice any patterns? Does your bowel program affect your bladder program? At what point did you contact your care provider? (If you contacted your care provider)	What were your first symptoms? Later symptoms? Was this sequence of symptoms usual for you? Do you notice any patterns? At what point did you contact your care provider? (If you contacted your care provider) Does your decision to contact your care provider differ for different episodes of urinary symptoms?
**Domain: Symptoms**	**Domain: Symptoms**	**Integrated Domain: Symptoms**
What UTI symptoms do you experience? Some common symptoms are: fever incontinence abdominal pain or burning cloudy appearance of urine blood in urine (pink urine) strong-smelling urine pelvic pain rectal pain low back pain increased spasticity autonomic dysreflexia.	Some common symptoms that may or may not mean people are getting/have a UTI: feeling unwell fever incontinence abdominal pain or burning cloudy appearance of urine blood in urine (pink urine) strong-smelling urine pelvic pain rectal pain low back pain increased spasticity autonomic dysreflexia urgency bladder spasms change in “regular” symptoms for people who always have some signs that, for them, don’t signal UTI different-from-normal sensation in some persons who use catheters other	Did we miss any symptoms that you experience? Identify Your Top 3: feeling unwell fever Incontinence/bladder spasms abdominal pain or burning ‘cloudy’ urine blood in urine (pink urine) strong-smelling urine pelvic pain rectal pain low back pain increased spasticity autonomic dysreflexia urgency change in “regular” symptoms that don’t always signal a UTI different-from-normal sensation other symptoms
**Domain: Bladder management techniques**	**Domain: Bladder management techniques**	**Integrated Domain: Bladder Emptying Routine History**
Do you have experience with UTIs and two or more different approaches to managing the bladder? Are there issues with managing UTI, facilitators or barriers, which you associate with different bladder management techniques? Have you changed your bladder management technique as time has gone on? How has your technique has evolved and why? How happy are you with what you are currently doing to manage UTI?	Do you empty your bladder through stoma or surgical opening? Do you use a single catheter once or multiple times? Do you tape the catheter in place for overnight? Do you ever clean the area prior to cathing? Does cathing empty the bladder and is there anything you must do to ensure the bladder gets emptied? If you have a cathing schedule, have you ever changed the frequency of cathing, such as from 3 to 2 hours?	What instruments and procedures do you use to empty your bladder? For example– Through a surgical opening (stoma) or urethra? Single use or cleansed and reused catheter? Schedule for emptying—Do you ever vary it? When? How do you make sure the bladder empties completely? Do you have a special procedure for nighttime while sleeping?
**Domain: Who treats your UTIs?**	**Domain: Dealing with Urinary Symptoms**	**Integrated Domain: Prescribed, preventative strategies and history**
Where do you go to get treatment for UTIs. For example, do you see your primary care physician, rrologist, or another specialist (ex. SCI doctor/physiatrist) to manage your UTIs? How many UTIs have you experienced in the last year that required antibiotics?	If you have an ongoing plan, was it self-initiated or prescribed by a healthcare provider? If so, what is that plan and with which provider is it established? PCP? Urologist? Physiatrist? Other? Why and when do you seek treatment from a provider? How do you determine which provider?	Do you have an on-going, preventive, bladder management plan? If so, what is it like? Did you initiate it or did your provider? Why and when do you seek treatment from a provider? How do you determine which provider? What has worked in the past but no longer works? When did this change occur? How many diagnosed UTIs have you had in the last year that required antibiotics? How was it diagnosed (e.g. presumptive, urinalysis, culture)? How does your bowel program affect your bladder program?
**Domain: Treatment for Urinary Symptoms?**	**Domain: Dealing with Urinary Symptoms**	**Integrated Domain: Selecting antibiotics and the role they currently play and have played in treatment**
How do you interact with your provider regarding taking antibiotics? How do you feel about taking them? How has your prescribed course changed over the years you have been managing UTI? Do you feel you experience any health changes related to taking antibiotics? How do you typically get your prescription? How do you typically work with your provider to monitor the progress of treatment? Do you maintain your own supply of antibiotics at home?	Do you get the opportunity to weigh in on the antibiotic prescribed? Do you take antibiotics that were not prescribed by a provider for the instance they are being taken? Do you take antibiotics prophylactically? If so, when? Do you have certain antibiotics you try to avoid? If so, which ones? What has worked in the past but no longer works? When did this change occur? At what point is the prescription given, when you are first seen or after the culture is resulted?	About your use of antibiotics… Are you given a prescription when you are first seen or after the culture results are known? Do you get the opportunity to help select the antibiotic prescribed? Do you take antibiotics that were prescribed by a provider for an earlier episode? Do you have a standing prescription from your provider for antibiotics? Do you take antibiotics prophylactically? If so, when? Do you have certain antibiotics you try to avoid? If so, which ones?
**Domain: Non-prescription strategies to decrease risk of UTI**	**Domain: Non-prescription strategies to manage urinary symptoms**	**Integrated Domain: Non-prescribed, preventative strategies and history**
Do you ever use non-prescription strategies prior to an antibiotic when you experience UTI symptoms? Do you ever use non-prescription and prescription strategies together? Have you shared your interest in/use of non-prescription strategies with your care provider? Where did you learn about non-prescription strategies?	Do you ever use non-prescription strategies prior to prescription medications when you experience urinary symptoms? Do you ever use non-prescription and prescription strategies together? Do you use frequency of fluid intake and bladder emptying (e.g. every 3 hours)? Do you make a point to avoid certain types of fluids or foods to reduce UTIs? Have you had experience with UTIs that did not require antibiotics? Did they go away on their own? Have you ever shared your interest in/use of non-prescription strategies with your care provider? Where did you learn about non-prescription strategies?	Have you developed routines on your own to curtail urinary symptoms? Have you tried any non-prescription strategies to manage urinary symptoms? For example … cranberry juice cranberry pills d-mannose probiotics increased fluid intake other strategies?
**Domain: Impact of UTIs on community participation and/or quality of life**	**Domain: Impact of urinary symptoms on community participation and/or quality of life**	**Integrated Domain: Impact of symptoms and treatment of symptoms on patient’s life**
How do UTIs influence your life? Do you engage in planning that you might not do if you didn’t experience chronic UTIs? Do you refrain from certain activities because of UTI?	Does antibiotic therapy cause disruptions to your daily routine or diet? Does it require special planning? Do you feel that urinary symptoms affect your planning (by increasing or decreasing the need to plan your schedule or other activities)? Do you refrain from certain activities or from planning activities in general because of urinary symptoms? Has fear of accidents, embarrassment due to odor, impacted how you participate in the community?	Increased need for planning Decision to not participate Embarrassment/fear of odor, accidents, etc. Other ways
**Domain: Insurance**	**Domain: Access to care & Financial Constraints**	**Integrated Domain: Logistics in attaining care**
Has insurance coverage influenced your bladder management approach? If yes, please describe how.	Do issues related to insurance or the process required to get attention from the medical community affect when you seek treatment? Has insurance coverage influenced your being able to use a catheter once each time you need it? Does the cost of culture and medication to treat UTI influence your care-seeking pattern? Is transportation, travel, and other logistics of getting to see a provider a factor in your decision to seek diagnosis/treatment? Is your first thought to self-treat, use a “home remedy” even if you HAVE insurance?	What factors (finances, time, travel, medication effects, etc.) determine whether or not you see a doctor when you have urinary symptoms? For example: What insurance covers and doesn’t cover Getting time off from work/school Negative impact from getting treatment, for example, diarrhea History of reflux (hydronephrosis) Others?

In Table 1, several features bear highlighting.

1. The integrated “triggers” domain comprised questions regarding pattern of symptoms and contacting your care provider in response to these patterns. Both perspectives began with a question regarding the first symptom. Patients suggest there may be a pattern experienced, whereas clinicians want to know if the patient has an internal (explicit or implicit) definition for ‘UTI.’ These questions seem to be getting at the same idea, an experience or pattern (patients) or threshold (clinicians) that suggests to the patient that they do or might have a UTI and so need to go get treatment.
2. The contributions from patients and clinicians in the integrated “symptoms” domain was perhaps the most closely aligned; both clinicians and patients suggested questions to learn about the symptoms and individual experiences with UTI. *All* respondents (clinicians and patients) conflated “UTI” and “urinary signs and symptoms.”
3. The integrated “bladder emptying history” domain was a focus for both clinicians and patients. The clinician’s questions used broad terms such as ‘bladder management technique’ whereas patients proposed questions of a more specific nature. An example of this is ‘Do you clean…. Do you tape the catheter…’ The perceived focus on changes in bladder management technique was removed in the final script, however, in order to maintain the respondents’ focus on what they do “now.”
4. The integrated “prescribed/history” domain tended to represent the clinicians’ perspective with its questions that focus on who treats UTIs and how frequently antibiotics were required. Patients presented questions on who treats UTIs and management plans. Both clinicians and patients focus on prescribed management plans.
5. The “selecting antibiotics” domain of the script was focused differently in the different perspectives. Specifically, patients propose questions focusing on the specific antibiotic that can or cannot be taken and when they are taken. Patients suggest the idea of taking antibiotics prophylactically, whereas clinicians ask if a supply of antibiotics is maintained by the patient. These questions diverge because the clinician’s question does not differentiate between prophylactically taking antibiotics and self-management of urinary symptoms with antibiotics.
6. The integrated “preventative” domain comprised questions in the same vein as comment 4, above (“prescribed/history”), but with a focus on strategies with non-prescribed compounds. Patients and clinicians each articulated that consulting a clinician is unnecessary to utilize these strategies. Once again, clinicians use verbiage that is quite broad, such as ‘non-prescription strategies’. Patients do this as well, but also included questions with more detail such as ‘frequency and fluid intake’.
7. The integrated “impact” domain represents the impact these experiences have on a person’s life. The patients focused on the impact of treatment on a person’s life, not just on their symptoms. Patients also suggested asking about the social anxiety/stress that may be caused by these events, which was not a consideration for the clinicians. However, queries from clinicians tended to be similar to those identified by the consumer experts.
8. The integrated “Logistics” domain reflects on the steps in obtaining care. The clinicians limited their questions to access, focusing on the impact that the patient’s insurance may have on it. Patients took a broader approach, from experiencing a symptom, to transportation, to insurance coverage. Finally, the patients ended their series of questions connecting these problems back to the potential to use ‘in-home’ treatments as a substitute.

The early clinical perspective shown in Table 1 included queries about: a) the relationship with, and confidence in, the provider; and b) how patients obtained their information/education/training with respect to UTIs and their management. CEs suggested elimination of these from the script, as they were not aligned with the purpose of the instrument in our study or for future research on treatments that might result in observable changes in the PC-PRO that this project sought to develop. The investigators agreed and left them out of successive versions of the script. Clinicians also focused on the treatable—not the most bothersome (or common)—signs and symptoms. These differences in particular underscore the importance of a phenomenologic approach (see Lasch et al.[22], Table 1)- even in an approach that blends phenomenologic and Grounded Theory methods, as we employed—to capturing the patients’ experience in the development of a *patient-centered* PRO. Moreover, these results from the development of the focus group script highlight how, while following the paradigms in Figs 1 and 2 would each lead to a patient-*reported* outcome being developed, the patient-*centeredness* of an instrument developed following the paradigm in Fig 2 would be greater. We used the integrated results from clinician and patient experts, refined by experience in the first three focus groups of patients and their caregivers, to complete the script that was used in the final 10 focus groups to inform the content of the urinary symptom instrument.

### Results: Development of the instrument: Patient focus groups

Thirteen patient focus groups, all conducted online for patients’ convenience, took place from December, 2014 to February, 2015. Each group was facilitated by two of the five CEs: one to facilitate and a second to pick up on subtleties for further probing that the facilitator may have missed. A member of the professional research team was on stand-by to help with technical difficulties should they arise. All focus groups were comprised of 3–5 participants, lasted 45–90 minutes, and were recorded and transcribed. Thematic analysis to identify and label signs and symptoms in a way consonant with patient experience was conducted by two trained CEs using NVivo10™ in consultation with a member of the research team with qualitative expertise (MMS). The results of the thematic analyses were discussed by all CEs, and their consensus was submitted to the research team.

Once clinician and patient results reached saturation, as discussed in the Methods section, the research team then iterated the results of the focus groups between the clinical and consumer experts. When their input ceased to change the item content, the beta version of the new urinary signs and symptoms instrument was created.

We then made the resulting instrument available online to obtain a nationally-representative sample of individuals with neuropathic bladder (NB) due to spinal cord injury. Responses from the first 300 respondents were analyzed with respect to the perspective of the item’s origins in order to further explore the differences in patient-centeredness of instruments that maintain the primacy of the patient perspective versus the clinical perspective.

### Results: Representative national sample responses to instrument

Based on the results of our instrument development procedure outlined above, we had a 29-item list of signs and symptoms comprising our PC-PRO, and we sought to validate it with a nationally representative sample of respondents. As noted, we had coded each item with respect to whether *or not* an item arose from a specifically patient-centered (PC) perspective. This coding was not included in the instrument itself but remained part of our data analysis plan.

**Fig 3:**
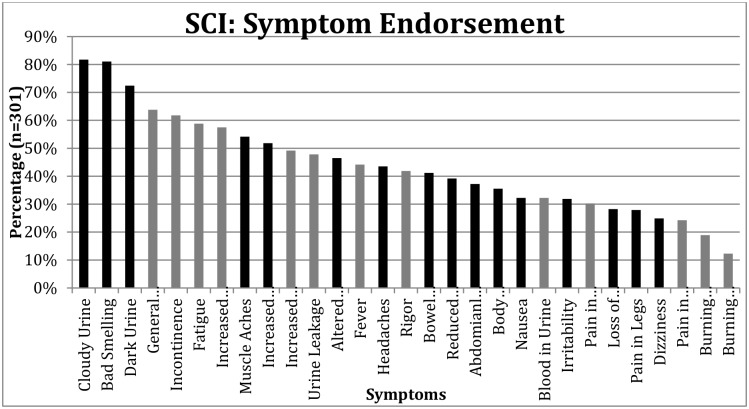
Endorsement rates (percent of 301 respondents) for signs and symptoms.

Of its 29 items, this instrument (the Urinary Symptom Questionnaire for people with Neuropathic Bladder, USQ-NB) included 13 signs/symptoms in the guidelines for diagnosing urinary tract infection set out by the Infectious Diseases Society of America (IDSA).[33] Our focus groups augmented these with 16 others, for a total of 29 signs and symptoms. Endorsement rates of our national sample on these 29 items were assessed (see Fig 3). The items that appear in the IDSA clinical practice guidelines are color-coded to show whether they represent those signs and symptoms included in clinical practice guidelines (CPGs),[33] shown in gray; items that were *not* included in the guidelines are shown in black. All items on the instrument represent signs/symptoms that were identified by our patient-led focus groups; the distinction in Fig 3 is that some of these items might not have been included if we had not encouraged the focus group participants to consider additional signs and symptoms beyond the CPGs.

Fig 3 shows that the three most commonly endorsed items on this instrument were identified for inclusion in the instrument by patients (black), and would not have been included if we had structured the instrument without explicitly incorporating the patients’ perspective. The three least-commonly endorsed items were submitted by the clinicians/derive from the clinical guidelines (gray). Seven of the 15 most frequently endorsed (42%-82% of our national sample), and six of the 14 least frequently endorsed items (10–40% endorsement), would have been missed if the patient perspective had not been explicitly and independently sought and maintained.

Table 1 suggests that if items had been added to the CPG[33] items (shown in gray in Fig 3) based solely on the input of our clinicians, we might have added items about access and relationship with provider. Table 2 explores the types of symptoms that were obtained from the patient perspective that are *not* included in the CPG[33].

**Table 2:** Origins of items on the USQ-NB: Patients or clinical practice guidelines[33].

Origin (PT/CPG) and type (urinary/general): USQ-NB Item:	Ur-PT	Ur-CPG	GEN-PT	GEN-CPG
Dark urine	1			
Blood in urine	1			
Cloudy urine	1			
Bad smelling urine		1		
Increased Frequency		1		
Increased Urgency		1		
Increased catheterization		1		
Increased incontinence	1			
Increased urine leak	1			
Reduced volume		1		
Pain in abdomen		1		
Pain low back	a		a	
Pain legs				1
Burning on catheterization			1	
Burning on passing urine		1		
Body position pain			1	
Fever			1	
Fatigue			1	
Generally not feeling well			1	
Muscle aches			1	
Altered sleep patterns				1
Irritability			1	
Headaches				1
Dizziness			1	
Rigor			1	
Abdominal bloating			1	
Nausea				1
Loss of Appetite				1
Bowel pattern change				1

Notes: USQ-NB = Urinary Symptom Questionnaire- Neuropathic Bladder. PT/CPG = Patient vs. Clinical Practice Guidelines (CPG). Ur-PT = Urinary symptom originating from Patients not included in CPG. Ur-CPG = Urinary symptom included in CPG (and also identified by Patients). GEN-PT = general physical symptom originating from Patients and not included in CPG. GEN-CPG = general physical symptom included in CPG (and also identified by Patients).

Table 2 shows that patients identified additional symptoms for inclusion in the USQ-NB that are both specifically urinary in nature (e.g., cloudy urine; increased incontinence) and also more general (e.g., fatigue; dizziness). Thus, their contributions to the instrument were not solely focused on one type of symptom. The instrument we developed (USQ-NB) includes queries about whether the patient feels the symptom is related to a UTI, but whether any of these is pre-infectious, infectious, or not related to an actual UTI is beyond the scope of this paper. Our research team is pursuing this line of investigation as we continue to document the utility of this new patient-centered PRO for research and clinical care in this domain.

## Discussion

The purpose of this study was to demonstrate, and then explore the result of, the use of a new paradigm for developing patient-*centered* patient reported outcomes (PC-PROs). This approach allows patients to report equally on items that are derived from both the patient and the clinician perspective–i.e., this new paradigm for developing patient*-centered*, patient-reported outcomes is fully consistent with the definition of a PRO from the FDA, the objectives of PCORI, and with most models of patient and stakeholder engagement. However, although valid patient-reported outcomes can be derived by either the dominant paradigm (Fig 1) or by the new paradigm (Fig 2), our results suggest that starting from—and maintaining—the *patient* perspective will promote the ‘patient-centeredness’ of the resulting instrument while still maintaining signs and symptoms that are clinically relevant. Focus groups and interviews that, for example, used only the IDSA guidelines for structure could easily have included an open-ended, “anything else?” item; instead we began the development of our scripts from the phenomenological perspective. The IDSA guidelines were published in 2009, so the identification of items from these guidelines was essentially *a priori*; the data we collected following our new model are new and support the conclusion that this new paradigm engages *patient and* clinician contributions around populating the list of signs and symptoms that patients in the target population encounter most frequently. Only by explicitly including, and then maintaining, the primacy of the patient perspective can the patient-*centered* dimension of a patient-reported outcome be retained/included.

The instrument is more than twice as long as it would have been without the active involvement of the patients; this may have implications for participant fatigue in some studies/populations. However, the focus group participants and our patient experts were enthusiastic about the opportunity to represent their own experiences fully. The number of items suggests that our paradigm is consistent with the PCORI intention “…to produce research that looks beyond the questions and measures found “under the lamp post,” within the researcher’s field of view”.[20]

This new paradigm is consistent with principles of “valuing the patient perspective”, and maintaining a “culture of patient centeredness in research”[14] while representing the rigorous qualitative methodology that constitutes the “crucial foundation” of PRO development.[22] When we have pilot intervention research to report on, the patient-centeredness of our instrument will facilitate our dissemination of our results. This emphasis on urinary signs and symptoms that are most commonly experienced, as well as those that are most clinically relevant, will support our further engagement of our stakeholders, while also promoting valid scientific research in the future. However, in our implementation of the new model, clinician input was obtained in focused meetings, and not focus groups, and the clinician participants were a single (convenience) sample of four individuals. While careful notes were obtained from all of these meetings, and. However, in future iterations in this line of study, we are planning to design the clinician input to derive from focus groups with a clinician who is trained in focus group facilitation, and to generally treat the clinician input the same way as the patients. We did not perceive it to be at the time, but this is a key feature of balancing the perspectives from clinicians and patients, so we have added this to the discussion of the new model.

The results suggest that the model of PRO development that we describe here and followed (Fig 2) can lead to PROs that maintain the patient perspective and are patient centered. We obtained substantial endorsement rates of all items in the resulting instrument by individuals across the United States, suggesting that each item is recognized as part of the lived experience with urinary symptoms in NB. However, there are limitations to consider. Firstly, we did not issue invitations to individual patients for the focus groups or for participation in our national beta test. Therefore, we cannot be sure of our response rate, or even how representative of persons with SCI or SB (or caregivers of persons with SB) who have NB these results are. We are currently preparing a second manuscript to discuss and describe the results of the survey, where this is discussed more fully. For the current study, the self-selected nature of responses to our survey is not a limitation with respect to the demonstration of how the new model for developing a PC-PRO (Fig 2) can lead to instruments that differ from the traditional model. We also have not included in this paper any formal discussion of the focus group participants; again, this is not relevant for a demonstration/proof of concept about how the PC-PRO development model works and yields a different PRO than would have been obtained without our focus on maintaining the primacy of the patient perspective.

Our results support the conclusion that there are qualitative differences in outcomes that are developed using an approach that maintains the primacy of the patient’s perspective–which we argue are concretely “patient-centered”, versus those that incorporate an emphasis on the patients’ report alone, consistent with the argument put forward by Basch, Abernethy & Reeve.[21] The PCORI Methods Committee [7] argued that “(t)o be truly patient-centered, PCOR faces the challenge of how to best incorporate the patient and caregiver perspective at every step in the selection of research questions, design, conduct, and analyses of studies, as well as dissemination and implementation of findings”[7]. Our paradigm establishes the primacy of the patient perspective in a PRO so that, once the instrument is validated, it will bring the patient and caregiver perspectives to every step of the design, conduct, and analyses of clinical research within the target domain. A recent systematic review [35] extensively reviewed the desirable characteristics of patient reported outcomes, and encapsulates how the traditional model (Fig 1) is deeply entrenched in the literature currently. In their synthesis of the literature and interviews with experts on the desirable characteristics of the PRO, claims of “patient-centeredness” are made but not supported, and no patient input was obtained—not in the literature that was reviewed and not in the interviewing that was conducted. Therefore, while our results (and the model here) *are consistent* with the characteristics that Francis et al. (2016)[35] discuss, the new PC-PRO development model is a clear departure from this traditional model; and these results suggest that the PC-PRO model does yield a qualitatively different instrument than a PRO would.

Overall, comparative effectiveness research that is conducted using a validated instrument developed using our paradigm will be stakeholder-driven [25] in that the instrument to be used as the outcome/endpoint in such research will be strongly representative of the patient/patient perspective. Moreover, the impact of maintaining the patient perspective on the design and interpretation of comparative effectiveness, and other types of clinical research, can be studied using an approach similar to what was described here. We are also continuing to study the psychometric characteristics of the instrument described here, including determining whether all items on the instrument are responsive to clinical change (e.g., as verified by biomarkers and metagenomic changes). Future work will also be needed to determine whether the development of PC-PRO instruments can work in domains where PRO instrumentation has been identified as problematic fields (e.g., radiology,[15, 16] emergency medicine,[17, 18] and neurosurgery).
